# Stimulus-dependent effects on tactile spatial acuity

**DOI:** 10.1186/1744-9081-1-18

**Published:** 2005-10-10

**Authors:** V Tannan, RG Dennis, M Tommerdahl

**Affiliations:** 1 Department of Biomedical Engineering, University of North Carolina at Chapel Hill, Chapel Hill, North Carolina 27599, USA

## Abstract

**Background:**

Previous studies have shown that spatio-tactile acuity is influenced by the clarity of the cortical response in primary somatosensory cortex (SI). Stimulus characteristics such as frequency, amplitude, and location of tactile stimuli presented to the skin have been shown to have a significant effect on the response in SI. The present study observes the effect of changing stimulus parameters of 25 Hz sinusoidal vertical skin displacement stimulation ("flutter") on a human subject's ability to discriminate between two adjacent or near-adjacent skin sites. Based on results obtained from recent neurophysiological studies of the SI response to different conditions of vibrotactile stimulation, we predicted that the addition of 200 Hz vibration to the same site that a two-point flutter stimulus was delivered on the skin would improve a subject's spatio-tactile acuity over that measured with flutter alone. Additionally, similar neurophysiological studies predict that the presence of either a 25 Hz flutter or 200 Hz vibration stimulus on the unattended hand (on the opposite side of the body from the site of two-point limen testing – the condition of bilateral stimulation – which has been shown to evoke less SI cortical activity than the contralateral-only stimulus condition) would decrease a subject's ability to discriminate between two points on the skin.

**Results:**

A Bekesy tracking method was employed to track a subject's ability to discriminate between two-point stimuli delivered to the skin. The distance between the two points of stimulation was varied on a trial-by-trial basis, and several different stimulus conditions were examined: (1) The "control" condition, in which 25 Hz flutter stimuli were delivered simultaneously to the two points on the skin of the attended hand, (2) the "complex" condition, in which a combination of 25 Hz flutter and 200 Hz vibration stimuli were delivered to the two points on the attended hand, and (3) a "bilateral" condition, in which 25 Hz flutter was delivered to the two points on the attended hand and a second stimulus (either flutter or vibration) was delivered to the unattended hand. The two-point limen was reduced (i.e., spatial acuity was improved) under the complex stimulus condition when compared to the control stimulus condition. Specifically, whereas adding vibration to the unilateral two-point flutter stimulus *improved *spatial acuity by 20 to 25%, the two-point limen was *not *significantly affected by substantial changes in stimulus amplitude (between 100 – 200 μm). In contrast, simultaneous stimulation of the unattended hand (contralateral to the attended site), *impaired *spatial acuity by 20% with flutter stimulation and by 30% with vibration stimulation.

**Conclusion:**

It was found that the addition of 200 Hz vibration to a two-point 25 Hz flutter stimulus significantly improved a subject's ability to discriminate between two points on the skin. Since previous studies showed that 200 Hz vibration preferentially evokes activity in cortical area SII and reduces or inhibits the spatial extent of activity in SI in the same hemisphere, the findings in this paper raise the possibility that although SI activity plays a major role in two-point discrimination on the skin, influences relayed to SI from SII in the same hemisphere may contribute importantly to SI's ability to differentially respond to stimuli applied to closely spaced skin points on the same side of the body midline.

## Introduction

Recently, we reported the development of a semi-automated method for measuring a human subject's ability to discriminate between two points on the skin [1]. In that study, a Two-Point Stimulator (TPS) was employed to deliver tactile stimuli simultaneously to two separate skin sites. Since distance between the two points of the TPS can be adjusted on a trial-by-trial basis, it was possible to employ a Bekesy tracking method to determine a subject's two-point limen under several different conditions of two-point stimulation. Two-point stimuli were presented to the skin under static conditions (two probes simply pressed into the skin), in the presence of flutter stimulation (probes oscillated at 25 Hz as they were pressed into the skin), or in the presence of vibration (probes oscillated at 200 Hz). The results duplicated the finding of Vierck and Jones [2] that demonstrated that oscillating the two probes improved a subject's spatial acuity (as measured by the two-point limen). Furthermore, both our study and the Vierck and Jones report showed that spatial acuity is better in the 25 Hz stimulus condition than in the 200 Hz stimulus condition.

Mountcastle and Darian-Smith [3] proposed that a subject's ability to spatially discriminate between two points on the skin would be dependent on the lateral inhibition that enables the formation of the peaks of neuronal activity in SI cortex. Additionally, LaMotte and Mountcastle [4,5] asserted that the capacity of a subject to accurately localize a flutter stimulus on the skin is determined by the locus and clarity of the flutter-evoked neuron population response within the topographically organized SI network. If this is the case, then the ability of a subject to discriminate between two points would improve if the locus of the responses in SI to the stimuli at the two corresponding skin sites were more clearly defined – i.e., if the spatial extent of the response in SI to a point stimulus waslimited or reduced. Observations by Tommerdahl and colleagues demonstrated that the SI response to a complex stimulus (one comprised of both flutter and vibration) is spatially constrained when compared to the response to flutter alone [6-9]. In other words, the SI response evoked by a complex stimulus is smaller in spatial extent than that evoked by 25 Hz flutter alone. Thus, based on the effect that same-site vibration has on the SI response to flutter, we were led to the prediction that vibration, if presented simultaneously at the same sites as two-point flutter stimuli (i.e., as a complex stimulus comprised of 25 Hz and 200 Hz components), would *improve *a subject's ability to discriminate between two points. Alternatively, recent findings comparing the SI activity evoked by different conditions of contralateral, ipsilateral and bilateral stimulation in the cat show that the magnitude of response in SI evoked by contralateral stimulation is reduced in the presence of an ipsilateral stimulus [10]. Similar results have been found in the non-human primate (unpublished observations). This led us to the prediction that, because of the decrease in prominence of the two peaks of neuronal activity in SI evoked and consequently, the reduction in the spatial clarity between those peaks of cortical activity, a subject's two-point limen would increase (indicating reduced spatial acuity) with the addition of a stimulus to the unattended hand.

## Results

**Figure 1:**
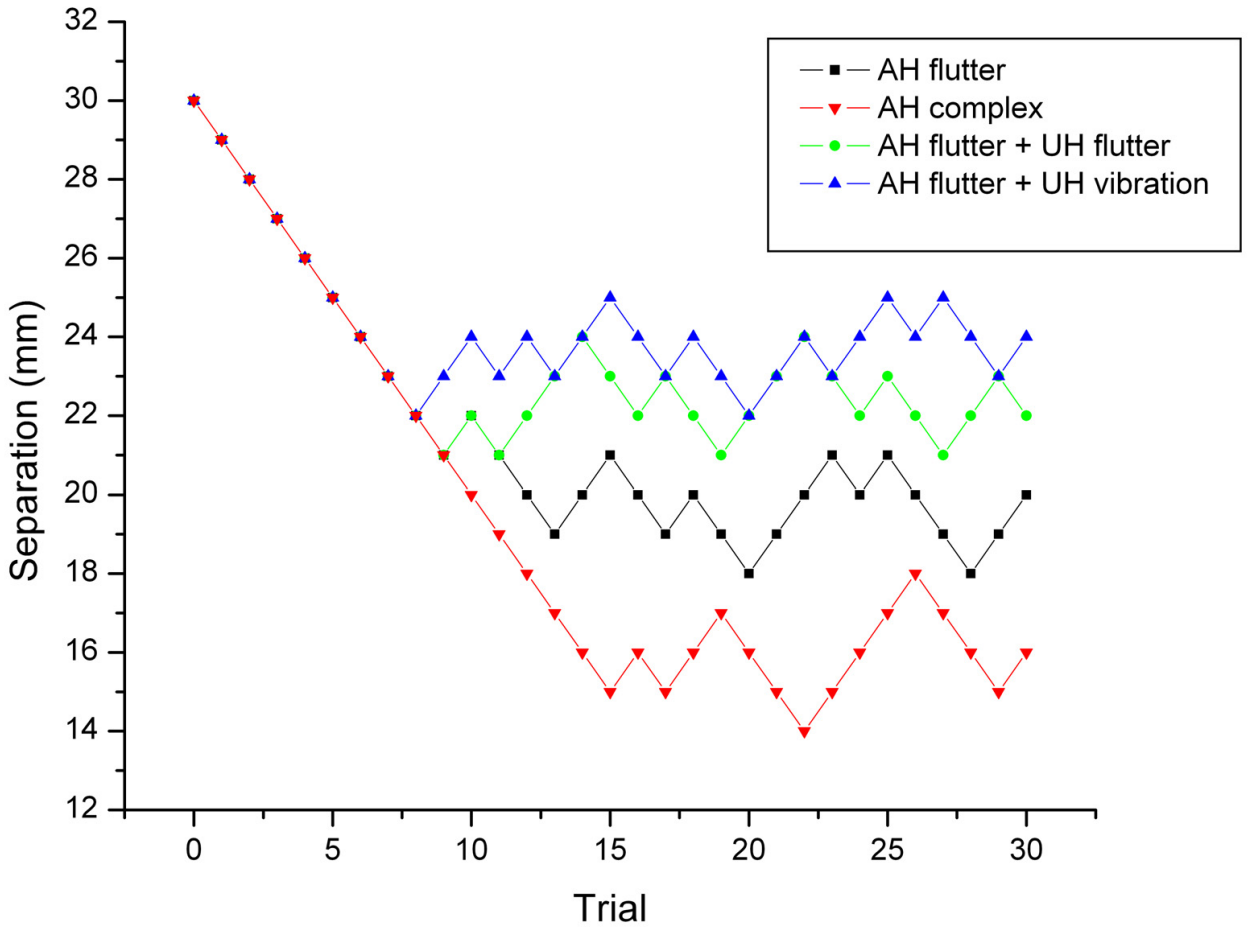
A tracking protocol was used to conduct a two-point limen threshold test. Separation between the two probe tips on the attended hand (AH) versus time was observed under four conditions of stimulation. One condition consisted of 25 Hz flutter applied by the TPS on the AH. In a second condition, the two tips were applied to the AH by a complex stimulus (25 Hz+200 Hz). For the other two conditions, 25 Hz flutter was applied to the AH with either a 25 Hz flutter or 200 Hz vibration stimulus applied simultaneously to the unattended hand (UH). A single trial consisted of stimuli presented to the skin for 1 sec, and then completely removed from the skin for an inter-stimulus interval of 2 sec. Each run consisted of 30 trials, or a duration of 90 sec total.

Bekesy tracking algorithms were used to find a subject's two-point limen at the dorsal surface of the right hand under four different stimulus conditions. Exemplary results for a single session (four runs) of a subject are shown in Figure 1. The two-point limen of the subject was tracked for two points delivered simultaneously and oscillated at 25 Hz on the attended hand (AH). The data presented indicate that under this condition the subject was able to detect the presence of two points at a separation of approximately 19 mm (average response for the last five trials). In a second run (the "complex" stimulus condition), the two-point limen was tracked under identical conditions as the first run, with the exception that the 25 Hz stimulus waveform was delivered with an additional 200 Hz vibration on the attended hand (see Methods). The addition of the 200 Hz vibration to the 25 Hz flutter resulted in a decrease in the two-point limen to approximately 16.4 mm. In the two other conditions, the two-point limen was tracked to a two-point 25 Hz flutter stimulus on the attended hand, under identical conditions as the first run, but with the addition of a simultaneous 25 Hz flutter or 200 Hz vibration stimulus to the opposite, unattended hand (UH). Interestingly, in both cases, stimulation of the unattended hand impaired the subjects' ability to discriminate between two points on the attended hand, and thus, the two-point limen actually increased to values of approximately 22 mm and 24 mm for 25 Hz and 200 Hz unattended conditions, respectively. To summarize, the detection of two points presented simultaneously with flutter was *improved *with same-site vibration and *degraded *with the addition of either a flutter or vibration stimulus on the opposite, unattended hand.

**Figure 2:**
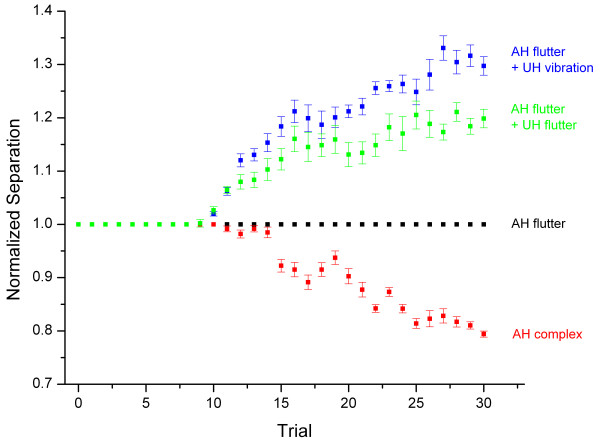
Average of two-point limen tracking, under all conditions, for one exemplary subject. All distances are normalized to the two-point distance recorded under the attended hand (AH) 25 Hz flutter condition. Standard error bars demonstrate that across-session variability for the two-point limen tracking method is fairly consistent.

To determine subject consistency of the above findings, the tracking data collected under each condition for an individual subject were averaged. The data were normalized to the flutter condition since the primary objective of this study was to determine the effect of vibration on the response normally evoked by two-point flutter stimulation. Thus, the two-point limen for the flutter condition was defined as the value "1" and all other distances are plotted as a proportion of the values obtained under the flutter condition [1]. The normalized average two-point limen plot for one subject is displayed in Figure 2. Note that the two-point limen was *reduced *(i.e., spatial acuity was improved) for the complex condition – the two-point limen tracks at approximately 80% of the values measured under the flutter condition. In contrast, the two-point limen was larger (i.e., spatial acuity is worse) for both bilateral conditions. In the case in which the opposite or unattended hand was presented with a simultaneous 25 Hz flutter stimulus, the two-point limen tracks approximately 20% higher than the control (attended hand only) condition. Similarly, applying a 200 Hz vibration stimulus simultaneously to the unattended hand resulted in two-point limen values that were approximately 30% higher than the control condition.

**Figure 3:**
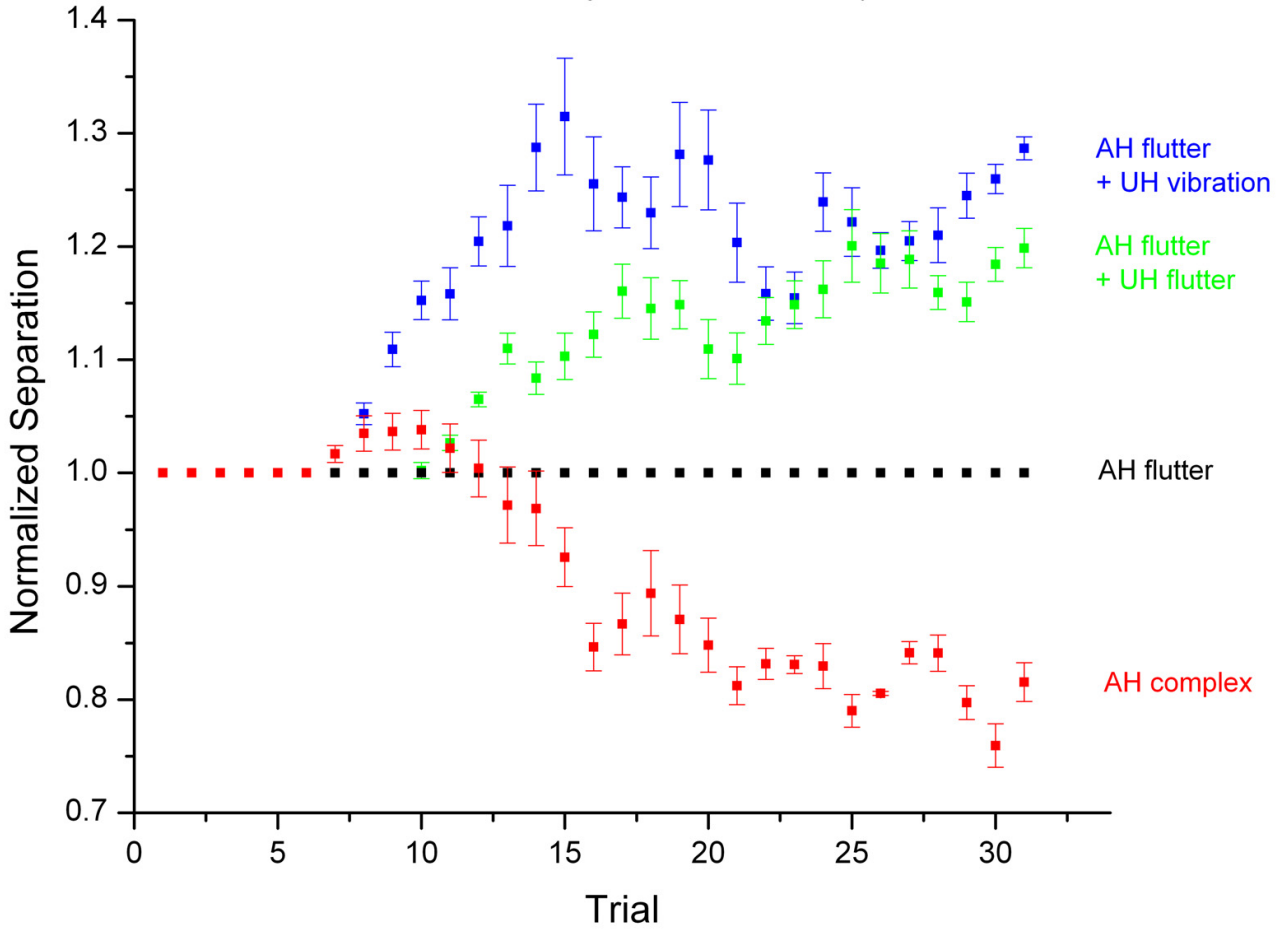
Average of two-point limen tracking across all subjects. All distances are normalized to the two-point distance recorded under the attended hand (AH) 25 Hz flutter condition. Standard error bars demonstrate that across-subject variability for the two-point limen tracking method is fairly consistent.

To determine the across-subject consistency of the above findings, the data normalization process applied to the single subject case, as shown in Figure 2 and described above, was repeated for data collected under each condition across all subjects. Normalized and averaged data are shown in Figure 3. Similar to the data presented in Figure 2, the two-point limen for the complex condition tracked between 75 and 80% of that measured under the flutter condition, indicating a 20–25% improvement in spatial acuity resulting from the presence of vibration during the flutter stimulus driving the TPS on the attended hand. Alternatively, tracking of the two-point limen showed an increase of approximately 20% and 30% for the conditions in which the unattended hand was stimulated with flutter and vibration, respectively.

**Figure 4:**
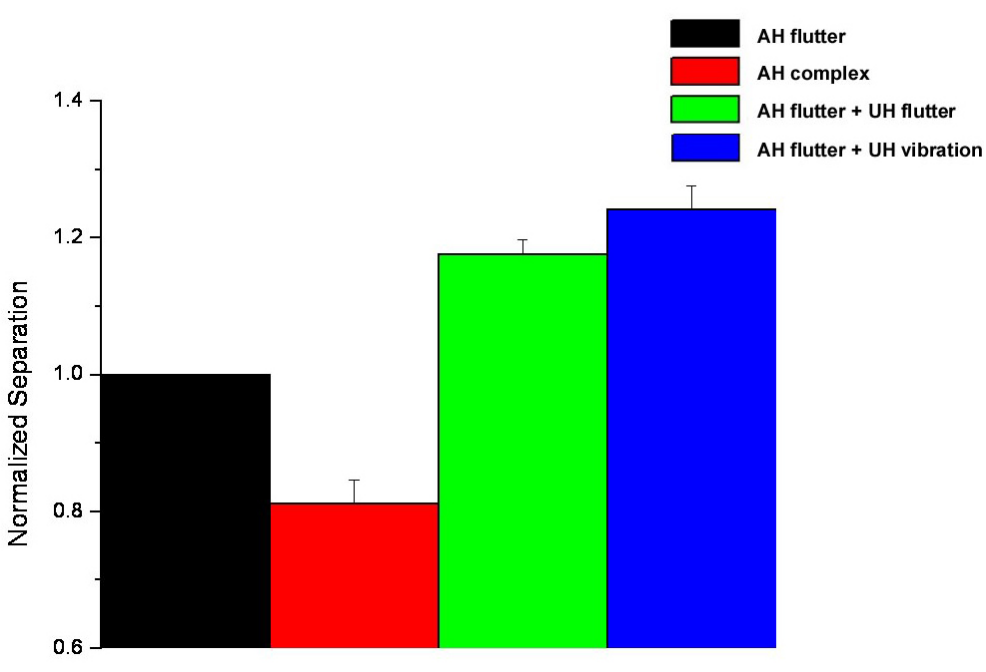
Average of last five trials of two-point limen tracking across all subjects, with standard error bars. All distances are normalized to the two-point distance recorded under the attended hand (AH) flutter condition.

In order to more directly compare the responses measured under each of the stimulus conditions, the tracking values obtained from the last five trials across all subjects was averaged and normalized to the flutter condition (Figure 4). Again, it is apparent that the two-point limen values decreased by approximately 20% under the complex condition, or when vibration was presented with flutter, by dual-site stimuli on the attended hand. Alternatively, the two-point limen increased when a second, simultaneous stimulus was added to the unattended hand – approximately 20% for the 25 Hz flutter condition and 30% for 200 Hz vibration condition. Standard error bars demonstrate that across-subject variability for the two-point limen tracking method is fairly consistent. ANOVA testing was conducted on this data with the null hypothesis that the mean under the control flutter condition is significantly different than the means obtained under the three test conditions. The means for the bilateral conditions of unattended hand 25 Hz (F = 47.7; p < 0.00000001) and unattended hand 200 Hz (F = 76.3; p < 0.00000001), as well as the complex condition (F = 27.6; p < 0.00001) are significantly different from the mean under the flutter condition.

**Figure 5:**
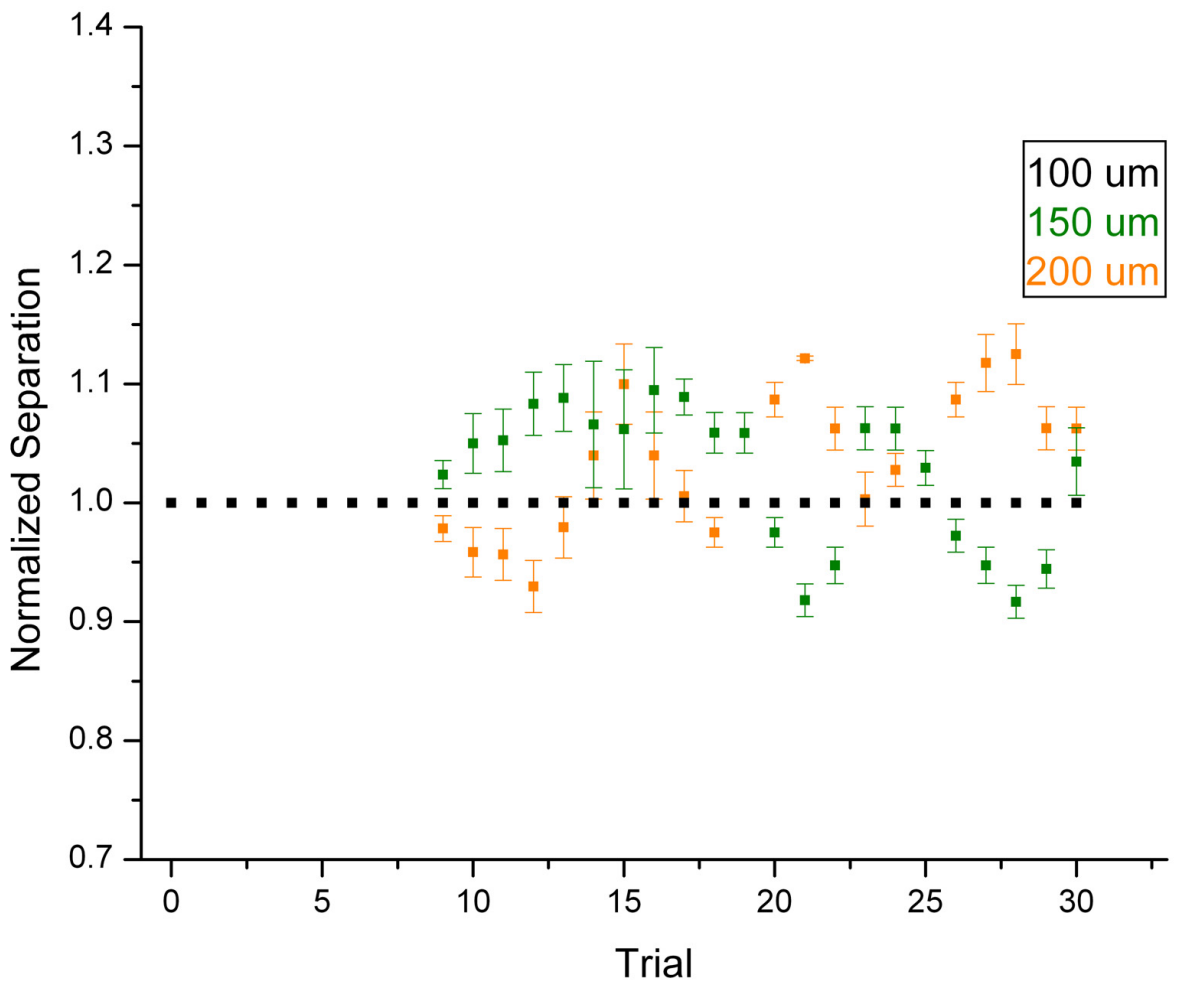
Average of two-point limen tracking across all subjects for control conditions: 25 Hz flutter stimulus applied by the TPS to the attended hand at amplitudes of 100 μm, 150 μm, or 200 μm. All distances are normalized to the two-point distance recorded under the 25 Hz-100 μm condition.

To ensure that the enhanced acuity of a subject under the complex stimulus condition was not due simply to the increased amplitude that resulted from adding vibration to a flutter stimulus (which resulted in a stimulus amplitude of 120 μm), the two-point limen was tracked on the attended hand at 25 Hz flutter of varying amplitudes. Specifically, a separate series of sessions were conducted to track and compare the two-point limen for the amplitudes of 100, 150, and 200 μm in the flutter-only condition. The results were normalized to the distances observed under the 100 μm condition and were plotted in the same manner as the previous results (see Figure 5). In both the 150 and 200 μm conditions, the two-point limen oscillated approximately within 10% of that observed at the 100 μm condition, suggesting that there was no consistent effect on the two-point limen due to the increased amplitude of the complex stimulus and that the effect seen under the complex condition was most likely attributable to the additional high-frequency component.

## Discussion

In the present study, we observed stimulus-dependent effects on two-point tracking of a flutter stimulus at the dorsal surface of the attended hand. The two-point limen was reduced (spatial acuity was improved) with a complex stimulus that consisted of 25 Hz flutter and 200 Hz vibration components. Specifically, it was found that adding vibration to the unilateral two-point flutter stimulus *improved *spatial acuity by 20 to 25%. When the amplitude of the unilateral two-point flutter stimulus was significantly varied (between 100 – 200 μm), the two-point limen was *not *affected. Simultaneous stimulation of the hand contralateral to the attended site, however, *impaired or reduced *spatial acuity by 20% with a flutter stimulus and 30% with a vibratory stimulus.

Vega-Bermudez and Johnson [11], using grating orientation studies, cited the importance of skin deformation as a factor affecting spatial acuity. For this reason, we considered the possibility that enhanced spatial acuity with a complex stimulus may be due to the fact that adding vibration to the flutter stimulus introduces another amplitude component, thereby increasing the overall magnitude of the stimulus. Results from our study showed that, within the amplitude range used, there were no significant differences in the two-point limen. This is also consistent with the idea that increasing amplitude of a stimulus does not increase the spatial extent of its cortical response – a finding recently reported [12]. In that report, observations obtained from imaging the optical intrinsic signal in non-human primates showed that higher amplitudes of stimulation with a 25 Hz flutter stimulus in the amplitude range studied (50–400 μm at a frequency of 25 Hz) did not produce larger areas of cortical activation in primary somatosensory cortex (SI). Rather, the spatial extent of the cortical patterns of activation evoked by the flutter stimulus was limited. Simons et al. postulated that the cortical response is sculpted or refined by lateral inhibition, thereby limiting changes in spatial extent [12]. These findings are consistent with the idea that the spatial extent of the SI response evoked by each of the point stimuli plays a role in a subject's ability to discriminate between two stimulus sites on the skin, since changing the amplitude of a flutter stimulus has little effect on either the spatial extent of the SI response in primates or on the two-point limen observations made in this report.

Summers and Chanter [13] reported results on tactile acuity in the fingertip in response to stimuli presented by a broadband tactile array. They found that localization of a 40 Hz target stimulus was improved with the addition of a 320 Hz background stimulus (which surrounded the target) compared to that with a 40 Hz background stimulus. However, Summers and Chanter also stated that this type of interpretation (that the addition of high-frequency vibration to a lower-frequency stimulus results in improvement in perception of that stimulus) was problematic because of the known differences between mechanoreceptors [13]. Previous studies had established the fact that spatial acuity was worse at high frequencies (in the Pacinian range) than at low frequencies (RA/SA range) [1,2,14]. However, if spatial acuity can be attributed to the spatial clarity between regions of cortical activity as LaMotte and Mountcastle [4] proposed, then the cortico-cortical interactions that result from the condition of simultaneous flutter and vibration [8] would undoubtedly have an effect on measures of spatial acuity. Flutter stimuli, such as the ones presented in this study, are known to evoke significant and sustained activity in SI cortex. Skin stimulation at 200 Hz, on the other hand, has been shown to reduce the spatial extent of SI response normally evoked by a 25 Hz flutter stimulus [6-9].

Tommerdahl et al. [6] compared the intrinsic signal evoked in areas 3b/1 by 25 Hz skin stimulation to the intrinsic signal evoked by a same-site skin stimulus containing both 25 and 200 Hz sinusoidal components (a "complex waveform stimulus"). Such experiments revealed that the increase in absorbance evoked in areas 3b/1 by a stimulus having both 25 and 200 Hz components was substantially smaller than the increase in absorbance evoked by "pure" 25 Hz stimulation of the same skin site. It was concluded that within a brief time after stimulus onset, 200 Hz skin stimulation evokes a powerful inhibitory action on area 3b/1 QA neurons. Inhibition due to same-site 200 Hz vibration may play a role in limiting the spatial extent of the cortical activity due to flutter stimulation, creating a sharper and more finely tuned response, suggesting improved spatial acuity.

The finding in previous OIS imaging experiments in cats that high-frequency skin stimulation is accompanied by a contralateral absorbance increase in area SII and, simultaneously, by a decline in absorbance in SI in the same hemisphere led Tommerdahl et al. [7] to consider the possibility that activity in the corticocortical connections that link SII with SI in the same hemisphere [15,16] leads to suppression/inhibition of SI during high-frequency skin stimulation. Insofar as the detailed mechanism by which SII might suppress/inhibit SI, the most straightforward possibility (first suggested by Hirsch and Gilbert) [17] is that long-range corticocortical (i.e., SII→SI) inhibition results from the distinct axonal termination patterns of the local inhibitory neurons in SI. That is, because the two major types of local inhibitory cells in the upper layers of somatosensory cortex (basket and chandelier cells) [18] terminate on cell bodies and initial segments of pyramidal cells, and either do not establish synaptic contacts with other inhibitory cells (this is the case for chandelier cells), or terminate only on the dendrites of inhibitory neurons (characteristic of basket cells), a strong excitatory input from another cortical area (e.g., the input that SI presumably receives from SII at only a brief delay after the onset of high frequency skin stimulation) should evoke an inhibitory process in the SI region that receives the upper layer input, and the inhibition should be selectively expressed on pyramidal cells.

A recent report described that the ability to localize a stimulus on the fingertips of one hand may be impaired with the interference of a similar stimulus on a fingertip of the opposite hand [19], suggesting that spatial acuity may be worse with bilateral stimulation than with unilateral stimulation under certain conditions. In a separate study, we reported that the SI cortical response to contralateral skin stimulation was reduced when an identical stimulus was presented simultaneously to the ipsilateral (mirror image) skin site [10]. Specifically, Tommerdahl and colleagues found that the magnitude of response in SI to bilateral stimulation was 30–35% smaller than the response evoked by a contralateral flutter stimulus. This finding led us to postulate that, since contralateral SI is recognized as the cortical region most responsible for spatial localization [4,5], a reduction in the magnitude of the contralateral SI response – via ipsilateral stimulation – could cause a reduction in spatial acuity. Results from the present study support this hypothesis, suggesting that bilateral stimulation of two homologous body parts leads to a decrease in the percept of spatial acuity.

**Figure 6:**
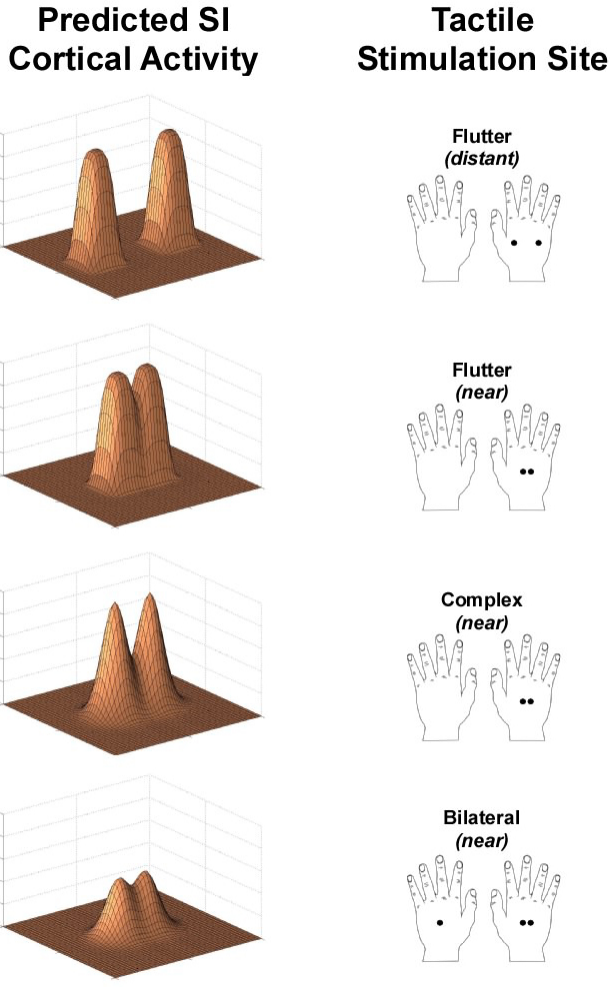
Model of predicted SI cortical activity in response to specific conditions of tactile stimulation. This model is an extension of the Vierck and Jones model (1970) on two-point receptive fields. a. When stimuli consisting of two points are oscillated on the skin at low-frequency 25 Hz flutter at distant sites, the peaks of SI response are distinct and non-overlapping, and therefore the subject is easily able to discriminate between the two points. b. As the points are positioned at stimulus sites that are closer together, the peaks begin to overlap. Because the peaks are no longer easily distinguishable, discriminability is reduced. c. Adding a same-site high-frequency 200 Hz vibration to the flutter stimuli ("complex" stimuli) has been shown to reduce the spatial extent of the peaks of response in SI and, as found in the present study, would make it easier to distinguish between two points on the skin. d. Presentation of a stimulus at the same skin site on the unattended hand would reduce the magnitude of SI response by flutter stimulation. This reduction in magnitude of SI response would consequently lead to a reduction in the clarity (or contrast) between the activity evoked by the adjacent, or near-adjacent, cortical regions activated by the two stimuli, and as a result, lead to a decrease in spatial acuity.

In a previously published report, Vierck and Jones [2] found that two-point discrimination is improved when the stimuli applied to the skin are oscillated versus static (not oscillated). Consequently, they proposed a model of how spatial acuity improved with oscillating versus static probes. In their report, Vierck and Jones [2] postulated that receptive fields in SI were smaller as a result of the oscillating stimulus condition, and that smaller receptive fields were less likely to overlap with one another, and thus, spatial acuity could improve as a result of changing stimulus conditions. We propose to extend that model by suggesting that the two-point limen is highly correlated with improvements in contrast between peaks of neuronal activity in SI that are evoked by stimulation of two adjacent or near-adjacent points on the skin. Figure 6 summarizes the effect that modification of the stimulus conditions, as reported in this paper, has on our proposed model of SI activity. It should be noted that this conceptual model has been influenced by recent findings about the SI cortical response to skin stimulation [8,10,12].

When stimuli consisting of two points are oscillated on the skin at low-frequency 25 Hz flutter at distant sites, the peaks of SI response are distinct and non-overlapping (Figure 6a). Thus, the subject is easily able to discriminate between the two points. As the points are positioned at stimulus sites that are closer together, the peaks of response begin to overlap (Figure 6b), and because the peaks of activity are no longer easily distinguishable, the two-point limen is increased (i.e., spatial acuity is worse). Adding a same-site high-frequency 200 Hz vibration to the flutter stimuli ("complex" stimuli) has been shown to reduce the spatial extent of the peaks of response in SI [6,8] (Figure 6c) and, as found in the present study, would make it easier to distinguish between two points on the skin. Presentation of a stimulus at the same skin site on the unattended hand would, predictably, reduce the magnitude of SI response by flutter stimulation [10]. This reduction in magnitude of cortical response would consequently lead to a reduction in the clarity (or contrast) between the activity evoked by the adjacent, or near-adjacent, cortical regions activated by the two stimuli (Figure 6d), and as a result, lead to a decrease in spatial acuity.

## Conclusion

In this paper, we propose a model that predicts a correlation between SI cortical activity and spatial acuity. Spatial acuity, as measured by the two-point limen, can be modified by changing stimulus conditions that would be predicted to have an impact on the SI cortical response. In particular, while vibration has the effect of reducing the spatial extent of SI cortical response normally evoked by flutter, such as when a vibrotactile stimulus comprised of both flutter and vibration is delivered to the skin, it also has the effect of improving a subject's ability to discriminate between two points on the skin. Presumably, this occurs as a result of vibration decreasing the spatial extent of the SI cortical response. Alternatively, stimulus conditions that are known to reduce the magnitude of the SI cortical response without changing the shape of response, such as when a second and simultaneous stimulus is delivered to a homotopic skin site on the opposite unattended hand, result in a reduction in spatial discrimination. While SI is regarded as playing a major role in two-point discrimination, this study provides evidence that other cortical areas that are connected to SI (such as SII) contribute importantly to SI's ability to differentially respond to closely spaced tactile stimuli.

## Materials & methods

Five naïve subjects (21–32 years in age) participated in this psychophysical study. All procedures were reviewed and approved in advance by an institutional review board.

Sinusoidal vertical skin displacement stimuli were delivered using the Cantek Metatron CS-525 vertical displacement stimulator (Cantek Metatron Corp., Canonsburg, PA). The stimulator made contact with the skin via the two tips of the Two-Point Stimulator (TPS) attachment (2.5 cm long, diameter 2 mm) fitted to the terminal end of the moving shaft of the stimulator transducer. The TPS is described in detail in a separate report [1]. An adjustable mechanical arm with lockable joints mounted to a free-standing, rigid platform (fabricated locally) enabled convenient adjustment and maintenance of stimulus position. A second identical Cantek stimulator, implemented in trials that required bilateral stimulation, was fitted with a single 2 mm diameter probe tip and positioned on the hand opposite the TPS in a similar fashion.

The subject was seated in a chair with arms placed comfortably on a table surface. Both arms were placed on X-ray bags filled with glass beads. The investigators molded the bags to fit the contours of the subject's arms, and when the subject was comfortable and the arms positioned appropriately to allow unimpeded access of the stimulator to the center of the dorsal surfaces of each hand, the bags were made rigid by evacuating them of air (achieved by connecting the bag to a vacuum line). In this way the arms were maintained in a comfortable but stable position for the full duration of the experimental session. The subject was unable to see either the experimenter or the stimulator and stimulus-control instrumentation. White noise presented via headphones eliminated potential auditory cues. A micrometer permitted the stimulator transducers and probe assembly to be lowered towards the predefined skin sites. The micrometer position at which the digital display on the stimulator controllers registered a 0.1–0.2 g change in resistive force was interpreted as the point at which the stimulator probes made initial contact with the skin.

A tracking protocol was used to conduct a two-point limen test, which determines the "least two-point separation at which the subject feels (has the subjective impression of) two points," [20] at the dorsal surface of the right hand. The hand dorsum was chosen because the innervation density at this site coincided with optimal resolution and separation capabilities of the TPS, and also because the surface is relatively flat, reducing confounds of skin curvature present at other potential sites of stimulation. Previous studies indicate that response to tactile acuity tests on the hand dorsum is similar to that on the fingertip, suggesting the dorsum to be a suitable site for such tests as well [21]. The subject was instructed to attend to the two-point stimulus presented by the TPS on the tested hand throughout experimentation. For each run, the two probe tips were initially spaced 30 mm apart. The stimuli were presented to the skin simultaneously for 1 sec at an indentation of 500 μm and then completely removed from the skin for 1 sec at an offset of -500 μm. The subject was given these two seconds to report feeling one or two points using a footswitch – no press for one point; a single press for two points. When two points were detected, the two probe tips moved closer together by a step (1 step = 1 mm); when only one point was detected, the two points moved farther apart by a step. The probe tips remained off the skin for the tip movement duration of 1 sec, thus the inter-stimulus interval lasted for a total of 2 sec. This process was repeated until a threshold could be determined, usually around 30 trials, hence a single run took approximately 90 sec. The inter-run interval was 60 sec in duration. The two-point limen was measured under four conditions of frequency and amplitude: unilateral 25 Hz-100 μm, unilateral 25 Hz-100 μm + 200 Hz-20 μm ("complex"), bilateral stimulation of 25 Hz-100 μm on both hands, and bilateral stimulation of 25 Hz-100 μm on the attended hand and 200 Hz-20 μm on the opposite unattended hand. In a session, four runs were conducted, each with one of the aforementioned stimulus conditions. In the bilateral conditions, stimuli were applied by a single timing mechanism and thus were presented to the skin in phase and synchrony. Order of stimulus conditions within a session was randomized and varied for each subject.

## Authors' contributions

VT conducted the experiments, analyzed the data and drafted the manuscript. RD had a role in the conduct and design of the experiments. MT was involved with the design of the experiments and the preparation of the manuscript.
